# Rheumatoid arthritis patients receive less frequent acute reperfusion and secondary prevention therapy after myocardial infarction compared with the general population

**DOI:** 10.1186/ar3151

**Published:** 2010-10-07

**Authors:** Sharon Van Doornum, Caroline Brand, Vijaya Sundararajan, Andrew E Ajani, Ian P Wicks

**Affiliations:** 1 Department of Medicine (RMH/WH), The University of Melbourne, Grattan Street, Parkville, 3050, Australia; 2 Department of Rheumatology, Royal Melbourne Hospital, Grattan Street, Parkville, 3050, Australia; 3 Clinical Epidemiology & Health Services Evaluation Unit, Royal Melbourne Hospital, Grattan Street, Parkville, 3050, Australia; 4 Centre for Research Excellence in Patient Safety, Monash University, Commercial Road, Melbourne, 3004, Australia; 5 Department of Medicine (Southern Clinical School), Monash University, Clayton Road, Clayton, 3168, Australia; 6 Department of Cardiology, Royal Melbourne Hospital, Grattan Street, Parkville, 3050, Australia; 7 NHMRC Centre of Clinical Research Excellence in Therapeutics, Department of Epidemiology and Preventive Medicine, Monash University, Commercial Road, Melbourne, 3004, Australia

## Abstract

**Introduction:**

The 30-day case-fatality rate after acute myocardial infarction (MI) for rheumatoid arthritis (RA) patients is twice that of the general population. This study compared the frequency and timeliness of early reperfusion therapy and treatment with secondary prevention medications after acute MI in RA patients and controls.

**Methods:**

We performed a structured medical chart review of RA patients and matched controls who had been admitted with acute MI to one of three hospitals in Victoria, Australia, between 1995 and 2005. The administration and timing of acute reperfusion therapy and in-hospital treatment with secondary prevention medications were compared between the two groups. Acute reperfusion was defined as thrombolysis or percutaneous coronary intervention (PCI) within 12 hours of the first symptom of MI.

**Results:**

The medical charts of 90 RA patients and 90 matched controls were reviewed. The RA patients were significantly less likely to receive acute reperfusion compared with the controls (16% versus 37%: odds ratio (OR), 0.27; 95% confidence interval (CI), 0.10 to 0.64)), and this difference persisted after adjusting for type of MI, clinical setting of MI, and prior MI (OR, 0.2; 95% CI, 0.05 to 0.6). The RA patients also received less-frequent in-hospital treatment with beta blockers (71% versus 83%; OR, 0.42; 95% CI, 0.18 to 0.96) and lipid-lowering agents (40% versus 70%; OR, 0.21; 95% CI, 0.09 to 0.46).

**Conclusions:**

RA patients who experience acute MI receive acute reperfusion and secondary prevention medications less frequently than do controls. This may contribute to higher case-fatality rates after MI in RA patients.

## Introduction

Rheumatoid arthritis (RA) is a chronic, systemic inflammatory disease that manifests primarily as inflammation in synovial joints. It is now widely recognized that RA patients also experience excess cardiovascular morbidity and mortality compared with the general population [1]. Not only do RA patients have an increased incidence of cardiovascular events, such as acute myocardial infarction (MI) [2-7], but they also have an increased case-fatality rate after acute MI compared with the general population [8-10]. For example, in our previous study of almost 30,000 individuals who had experienced a first acute MI, the adjusted odds ratio (OR) for 30-day mortality in RA patients versus controls was 1.9 (95% confidence interval (CI), 1.3 to 2.7) [8].

The reasons for a higher case-fatality rate after MI in RA patients are not known. Possible explanations include delays in seeking medical attention or in diagnosis, detrimental effects of systemic inflammation or concurrent RA medications or both, different patterns of coronary artery disease in RA patients, or differential treatment after the acute cardiac event. In our aforementioned study, we found that percutaneous coronary intervention (PCI) was performed half as often in RA patients as in the controls (adjusted OR, 0.5; 95% CI, 0.3 to 0.8), which suggests that RA patients receive differential treatment [8]. However, in that study, we had no data on the frequency of administration of thrombolysis, the reason(s) that PCI was less frequently used in the RA patients, nor the frequency of treatment with secondary prevention pharmacotherapy after MI.

The aim of the present study was to investigate whether the treatment received by RA patients after an acute MI is different from that received by the general population, and if so, why. We addressed this by comparing the frequency of acute reperfusion therapy (PCI or thrombolysis) and treatment with secondary prevention pharmacotherapy in RA patients compared with controls after acute MI.

## Materials and methods

We performed a structured medical chart review of RA patients and matched controls who had been admitted with acute MI to one of three hospitals in Victoria, Australia (The Royal Melbourne Hospital, The Alfred Hospital, and Epworth Hospital) between 1995 and 2005. The Royal Melbourne and Alfred hospitals are major metropolitan public hospitals, and the Epworth is a major metropolitan private hospital. The study was approved by the ethics committee of each of the participating hospitals. Informed consent from patients was not required by the ethics committees, as the study involved chart review only.

### Identification of study population

We obtained from each hospital a de-identified list of all patients with a discharge diagnosis of acute MI (International Classification of Diseases (ICD) 9-CM (Aust v2) code 410.xx or ICD 10-AM code I21.xx) between 1 July 1995 and 30 June 2005 (hereafter referred to as "MI admission"). The 15,278 individuals had received a discharge diagnosis of acute MI from one of the three participating hospitals between 1 July 1995 and 30 June 2005. We identified RA patients as those who had also received a discharge diagnosis code for RA (ICD 9-CM (Aust v2) code 714.xx or ICD 10-AM codes M05.xx, M06.xx, or M08.0) during the MI admission or any previous admission. Within the cohort of 15,278 acute MI cases, 133 individuals had also received a discharge diagnosis of RA and were classified as RA cases. The remaining 15,145 patients were classified as non-RA. The medical charts of the 133 RA patients were requested for formal review. Forty-three (32%) of the RA cases were not able to be included in the study: nine (7%) of the charts were unavailable or incomplete, 20 (16%) did not meet criteria for RA, and 14 (11%) did not meet criteria for definite MI. The remaining 90 RA patients (50, Alfred Hospital; 25, Royal Melbourne Hospital; 15, Epworth Hospital) were included in the study. The chart review of the control patients was performed in a similar manner. Thirty-four (38%) of the 90 matched controls initially selected were not able to be included in the study: 18 (20%) charts were unavailable or incomplete, and 16 (18%) patients did not meet criteria for definite MI. In these instances, a second matched control was selected from the de-identified list.

### Definitions used for the chart review

The diagnosis of RA was considered to be correct if a doctor had documented the diagnosis of RA in the chart during the MI admission or during any previous hospital encounter. We chose this definition of RA because comprehensive documentation of RA diagnostic criteria is frequently omitted in hospital medical notes, especially if the patient has been admitted with a nonrheumatologic medical condition (such as MI). We predicted that the use of more-stringent diagnostic criteria would result in the exclusion of many patients who truly had RA, because of an absence of detailed medical notes, rather than any doubt about the diagnosis. For each patient who met our definition of RA, we recorded additional information supporting the diagnosis of RA, including whether the patient fulfilled American College of Rheumatology (ACR) diagnostic criteria [11] or was being treated with disease-modifying antirheumatic drug (DMARD) therapy or both.

Myocardial infarction was defined according to the 2003 American Heart Association Scientific Statement [12] and was categorized as either ST-elevation MI (STEMI) or non-STEMI. We included only patients who were classified as "definite" MI. We defined "acute reperfusion" as the administration of thrombolysis or PCI within 12 hours of the first symptom of MI. Preexisting cardiovascular risk factors and comorbidities were recorded if they were documented in the medical chart or if the patient was receiving treatment for the condition during the MI admission. The documentation of a medical condition in the chart was largely due to patient self-report; however, results of prior investigations (for example, angiography, surgical procedures) or prior hospital admissions or both were also taken into consideration. Medications that we considered indicative of a medical condition included lipid-lowering agents (for hypercholesterolemia) and oral hypoglycemic agents or insulin (for diabetes mellitus).

### Chart-review procedure

The RA patients' charts were first reviewed for confirmation of the diagnoses of RA and MI, as defined earlier. If the diagnoses of RA and MI were not confirmed, the patient was excluded from the study. If the diagnoses of RA and MI were confirmed, the patient was included in the study, and a full review of the chart was performed, as described later. For each RA patient in the study, a non-RA control matched for age at time of MI (± 1 year), gender, treating hospital, and year of MI (± 1 year) was selected from the de-identified list. The control patients' charts were reviewed for a diagnosis of MI, as defined earlier. If the diagnosis of MI was not confirmed, the patient was excluded from the study, and another control was selected from the de-identified list. If the diagnosis of MI was confirmed, the patient was included in the study, and a full review of the chart was performed.

Chart abstraction and data collection were performed by a trained research assistant using a structured audit form. The accuracy of the chart abstraction was verified by independent audit of 5% of the charts by a second investigator (SV). The RA patients' charts were reviewed for RA-related characteristics, including ACR diagnostic criteria [11], disease duration, extraarticular disease, and DMARD treatment in the past and at the time of the MI admission. The charts of all patients (RA and non-RA) were reviewed for: confirmation of MI and type of MI, conventional cardiovascular risk factors and other comorbidities (including prior MI), medications taken at time of MI admission, administration and timing of reperfusion therapy (thrombolysis or PCI), and post-MI events such as in-hospital death and coronary artery bypass graft surgery. The clinical setting of the MI was classified into one of three categories: (a) "community based," the MI occurred outside a hospital with no acute medical illness immediately preceding it; (b) "after medical illness," the patient was already an inpatient for a medical illness and experienced the MI as a complication during that hospital admission; or (c) "after surgical event," the patient was already an inpatient and had undergone a surgical procedure and experienced the MI as a complication during that hospital admission. Urgent reperfusion is not necessarily indicated in all cases of MI; however, in the event of STEMI, early thrombolysis or PCI is considered the standard of care [13]. In those patients who experienced an STEMI and did not receive acute reperfusion, the reason for lack of acute reperfusion was determined from extensive review of the chart and was categorized as either (a) delayed diagnosis due to late presentation; (b) delayed diagnosis due to diagnostic uncertainty; (c) presence of a contraindication; or (d) unable to ascertain the reason. We also recorded whether treatment with secondary prevention medications (specifically aspirin, beta blockers, angiotensin-converting enzyme (ACE) inhibitors, lipid-lowering agents, or a combination of these) was commenced during the hospital stay for each patient. If a patient had an allergy or other condition precluding the use of these medications, or if the patient was previously taking the medication and it was continued during the MI admission, the treatment was considered to have been provided.

### Statistical analysis

The primary end point was the proportion of patients who received acute reperfusion after MI. In performing our sample-size calculation, we used data from our previous study, in which we found that RA patients were treated with PCI half as often as non-RA patients. Assuming acute reperfusion rates of 50% in the control patients and 25% in the RA patients, with power = 0.9 and alpha = 0.05, we calculated that a sample of 85 participants per group would be required. Descriptive data were summarized by using population means and standard deviation or percentages, as appropriate. Baseline characteristics and treatment with secondary prevention medications of the RA and control patients were compared by using the McNemar test for categoric variables and the paired *t *test for continuous variables. Treatment with cardiac interventions such as acute reperfusion, thrombolysis, and PCI in the RA and control patients was compared with the McNemar test (unadjusted analysis) and with conditional logistic regression adjusted for clinical setting of MI, type of MI, and prior MI. Our study covered a 10-year period during which the treatment of acute coronary syndromes evolved considerably. We therefore performed a *post hoc *analysis in which we compared treatment with acute reperfusion and secondary prevention pharmacotherapy between RA and controls over three time periods: prior to 1999, 1999 to 2002 inclusive, and after 2002. Analyses were performed with the use of Stata software version 10.0 (StataCorp, College Station, TX, USA).

## Results

Demographic details, cardiovascular risk factors, and other comorbidities of the RA and control patients are shown (Table 1). Fifty-five (61%) of the patients were women, with a mean age of 71 ± 10 years. The RA group had longstanding disease (mean disease duration, 20 ± 13 years) and were taking an average of 1.4 DMARDs at the time of the MI. Only one RA patient was using a biologic DMARD. Forty-nine (54%) of the RA patients had sufficient documentation in the chart to confirm the diagnosis of RA according to ACR criteria. Seventy-eight (87%) of the RA patients either fulfilled ACR criteria or were taking one or more DMARDs at the time of the MI.

**Table 1:** Demographic and clinical features of the RA and control patients admitted to participating Victorian hospitals with acute MI between 1995 and 2005

	RA (*n *= 90)	Controls (*n *= 90)
Female, *n *(%)	55 (61)	55 (61)
Age in years, mean (SD)	71 (10)	71 (10)
RA disease duration in years, mean (SD)	20 (13)	-
Seropositive, *n *(%)^a^	35 (78)	-
No. of DMARDs, mean (SD)	1.4 (1.1)	-
Taking NSAID at time of MI, *n *(%)	21 (23)	6 (7)^b^
Preexisting comorbidities:		
Ischemic heart disease, *n *(%)	31 (34)	33 (37)
Prior MI, *n *(%)	10 (11)	11 (12)
Cerebrovascular disease, *n *(%)	8 (9)	9 (10)
Peripheral vascular disease, *n *(%)	19 (21)	5 (6)^b^
IDDM, *n *(%)	3 (3)	1 (1)
NIDDM, *n *(%)	22 (24)	27 (30)
Hypertension, *n *(%)	52 (58)	56 (62)
Hypercholesterolemia, *n *(%)	22 (24)	38 (42)^b^
Current smoker, *n *(%)	14 (15)	16 (18)
Previous smoker, *n *(%)	41 (46)	27 (30)
Congestive cardiac failure, *n *(%)	15 (17)	11 (12)
Chronic lung disease, *n *(%)	28 (31)	16 (18)
Chronic renal impairment, *n *(%)	15 (17)	8 (9)

Chronic lung disease: asthma, chronic obstructive airways disease, and pulmonary fibrosis; DMARD, disease-modifying antirheumatic drug; IDDM, insulin-dependent diabetes mellitus; MI, myocardial infarction; NIDDM, non-insulin-dependent diabetes mellitus; NSAID, nonsteroidal antiinflammatory drug; RA, rheumatoid arthritis. ^a^Data available for 45 of 90 RA patients. ^b^*P *value < 0.05.

No difference was found in the prevalence of traditional cardiovascular risk factors between the two groups, with the exception of hyperlipidemia, which was less prevalent in the RA group than in the control group (24% versus 42%; *P *= 0.02). The prevalence of ischemic heart disease, prior MI, and cerebrovascular disease was similar in the two groups; however, peripheral vascular disease was more common in the RA group than in the control group (21% versus 6%; *P *= 0.002).

### Reperfusion therapy

Table 2 shows the treatment received by the RA and control patients after acute MI. No difference was noted between hospitals in the proportion of patients who received acute reperfusion (data not shown), so we present combined hospital data. In the unadjusted analysis, the RA patients were significantly less likely to receive acute reperfusion compared with the controls (16% versus 37%, respectively; OR, 0.27; 95% CI, 0.10 to 0.64). Thrombolysis was performed in 9% of the RA patients compared with 24% of the controls (OR, 0.3; 95% CI, 0.1 to 0.77); PCI was performed in 11% of the RA patients compared with 33% of the controls (OR, 0.2; 95% CI, 0.06 to 0.53); and coronary angiography was performed in 43% of the RA patients compared with 58% of the controls (OR, 0.41; 95% CI, 0.16 to 0.92). Coronary artery bypass surgery was performed more frequently in the controls; however, this variance did not reach statistical significance.

**Table 2:** Treatment received by RA and control patients after myocardial infarction

	RA (*n *= 90)		Controls (*n *= 90)					
						
	***n***	%	***n***	%	**Unadjusted OR**^**a**^ (95% CI)		**Adjusted OR**^**b**^ (95% CI)	
Acute reperfusion^c^	14	16	33	37	**0.27**	**(0.10-0.64)**	**0.21**	**(0.07-0.62)**
Thrombolysis	8	9	22	24	**0.30**	**(0.10-0.77)**	**0.33**	**(0.11-0.96)**
PCI	10	11	30	33	**0.20**	**(0.06-0.53)**	**0.23**	**(0.09-0.63)**
PCA	39	43	52	58	**0.41**	**(0.16-0.92)**	0.54	(0.23-1.25)
CABGS	11	12	17	19	0.57	(0.21-1.46)	0.67	(0.27-1.65)

CABGS, coronary artery bypass graft surgery; CI, confidence interval; OR, odds ratio; PCA, percutaneous coronary angiography; PCI, percutaneous coronary intervention (angioplasty ± insertion of stent); RA, rheumatoid arthritis. **^a^**McNemar χ^2 ^test. ^b^Conditional logistic regression, adjusting for type of MI (STEMI or NSTEMI), presence of prior MI, and clinical setting of MI. ^c^Acute reperfusion defined as the administration of thrombolysis or PCI within 12 hours of the first symptom of MI.

The presence of a medical or surgical illness immediately before the MI was substantially more common in the RA patients than in the controls (31% versus 16%, respectively: OR, 2.5; 95% CI, 1.2 to 5.5). The clinical setting of the MI, the type of MI, and the past history of MI could potentially have affected the likelihood that acute reperfusion was provided. Therefore, we performed an analysis by using conditional logistic regression, with adjustment for these variables (Table 2). In the adjusted analysis, RA patients remained significantly less likely to be treated with acute reperfusion, thrombolysis, and PCI, suggesting that acute concurrent conditions or prior MI or both were not responsible for the observed differential treatment of RA patients.

We also performed an analysis restricted to the subgroup of RA patients who fulfilled ACR criteria (*n *= 49) along with their matched controls. Compared with the controls, the ACR-defined RA patients were significantly less likely to receive acute reperfusion (OR, 0.31; 95% CI, 0.11 to 0.85), thrombolysis (OR, 0.14; 95% CI, 0.03 to 0.62), and PCI (OR, 0.27; 95% CI, 0.07 to 0.98). Coronary angiography and coronary artery bypass surgery were performed more frequently in the controls; however, this variance did not reach statistical significance.

### Characteristics of patients not receiving reperfusion therapy

From our total study cohort of 180 patients, 29 of the RA patients and 39 of the controls were diagnosed with STEMI. Acute reperfusion was administered in 48% of the RA-STEMI patients versus 74% of the control-STEMI patients (*P *= 0.027 with the χ^2 ^test). In those STEMI patients who did not receive acute reperfusion (*n *= 25), delayed diagnosis of MI was the most common reason for failure to provide acute reperfusion: six (40%) of RA patients and five (50%) of controls. It was previously demonstrated that RA patients experience a higher rate of silent MI than does the general population, and this may also contribute to a delay in presentation to hospital [9,14]. In our study, 39% of the RA patients did not experience chest pain during the MI compared with 22% of the control patients (OR, 0.5; 95% CI, 0.3 to 0.9). A contraindication to acute reperfusion was present in five (33%) of the RA patients, whereas none of the controls had a contraindication. In four (27%) of the RA patients and five (50%) of the controls, no clear reason was found for not providing acute reperfusion after careful review of the medical chart.

The numbers were too small in this subgroup of 25 patients to make any meaningful statistical comparisons; however, the difference in the prevalence of contraindication to acute reperfusion between the RA and control groups was of interest. Five RA patients had a contraindication to acute reperfusion; one had peptic ulcer disease (bleeding risk), one had a recent intracerebral bleed, and three patients had undergone major surgery in the previous 5 days. This was consistent with our finding that an antecedent medical illness or surgical event was more common in the RA subjects.

### Secondary prevention therapy

Table 3 shows the number of RA and control patients who received treatment with selected secondary prevention medications. The RA patients received less-frequent treatment with beta blockers (71% versus 83%: OR, 0.42 (95% CI, 0.18 to 0.96)) and lipid-lowering agents (40% versus 70%: OR, 0.21 (95% CI, 0.09 to 0.46)).

**Table 3:** In-hospital treatment with secondary prevention medications received by RA and control patients after myocardial infarction

	RA (*n *= 90)		Controls (*n *= 90)			
				
	***n***	%	***n***	%	**OR**^**a **^**(95% CI)**	
Aspirin	85	94	89	99	0.20	(0.02-1.71)
Beta blockers	64	71	75	83	**0.42**	**(0.18-0.96)**
ACE inhibitors	61	68	57	63	1.18	(0.67-2.08)
Lipid-lowering agents	36	40	63	70	**0.21**	**(0.09-0.46)**

ACE, angiotensin-converting enzyme; CI, confidence interval; OR, odds ratio; RA, rheumatoid arthritis. **^a^**McNemar χ^2 ^test. Acute reperfusion is defined as the administration of thrombolysis or PCI within 12 hours of the first symptom of MI.

Ten of the RA patients and 11 of the controls had experienced MI before the study "MI admission." Table 4 shows the numbers of these patients who were already taking selected secondary prevention medications at the time of the study MI. The RA patients had lower rates of treatment with aspirin, beta blockers, and lipid-lowering agents at the time of their recurrent MI. These data mirror our findings in the analysis of the entire cohort and serve to validate the results of the present study.

**Table 4:** Use of secondary prevention medications at the time of "MI admission" in RA and control patients with a prior history of MI

	RA (*n *= 10)	Controls (*n *= 11)
Aspirin, *n *(%)	3 (30)	6 (55)
Beta blocker, *n *(%)	1 (10)	4 (36)
ACE inhibitor, *n *(%)	3 (30)	3 (27)
Lipid-lowering agent, *n *(%)	2 (20)	5 (45)

ACE, angiotensin-converting enzyme; RA, rheumatoid arthritis.

### Changes in post-MI care over time

Table 5 shows the frequency of selected post-MI treatments in RA and control patients for three time periods within our study period: prior to 1999, 1999 through 2002, inclusive, and 2003 and onward. For virtually all interventions, in each of the time periods, the RA patients received less-frequent treatment than did the controls. Within the subgroup of STEMI patients, a suggestion of higher rates of acute reperfusion was found in the most recent time period (small numbers limit this result); however, in all time periods, the RA patients received less-frequent acute reperfusion than did the controls. Treatment with lipid-lowering agents increased progressively over time for all patients; however, in each time period, RA patients again received less-frequent treatment with lipid-lowering agents than did controls.

**Table 5:** Post-MI treatment in RA and control patients: comparison of three time periods

	Prior to 1999	1999 to 2002 inclusive	2003 onward
Acute reperfusion (all patients)			
RA patients, *n *(%)	6/16 (38%)	4/38 (10%)*	4/28 (12%)
Controls, *n *(%)	10/19 (53%)	17/41 (41%)	6/30 (20%)
Acute reperfusion (STEMI patients)			
RA patients, *n *(%)	6/10 (60%)	4/13 (31%)*	4/6 (67%)
Controls, *n *(%)	9/12 (75%)	14/20 (70%)	6/7 (86%)
Thrombolysis			
RA patients, *n *(%)	3/16 (19%)	3/42 (7%)*	2/32 (6%)
Controls, *n *(%)	8/19 (42%)	12/41 (29%)	2/30 (7%)
Percutaneous coronary intervention			
RA patients, *n *(%)	4/16 (25%)	3/42 (7%)*	3/32 (9%)*
Controls, *n *(%)	3/19 (16%)	15/41 (36%)	12/30 (40%)
Aspirin			
RA patients, *n *(%)	14/16 (88%)	41/42 (98%)	30/32 (94%)
Controls, *n *(%)	19/19 (100%)	41/41 (100%)	29/30 (97%)
Beta blockers			
RA patients, *n *(%)	8/16 (50%)	32/42 (76%)	24/32 (75%)
Controls, *n *(%)	15/19 (79%)	32/41 (78%)	28/30 (93%)
ACE inhibitors			
RA patients, *n *(%)	8/16 (50%)	29/42 (69%)	24/32 (75%)
Controls, *n *(%)	14/19 (74%)	24/41 (58%)	19/30 (63%)
Lipid-lowering agents			
RA patients, *n *(%)	0/16 (0)*	16/42 (38%)*	20/32 (62%)
Controls, *n *(%)	7/19 (37%)	31/41 (76%)	25/30 (83%)

ACE, angiotensin-converting enzyme; MI, myocardial infarction; RA, rheumatoid arthritis; STEMI, ST-elevation myocardial infarction. *P < 0.05 for the comparison with control patients.

## Discussion

We previously demonstrated an almost twofold increase in the 30-day case-fatality rate after MI in RA patients; however, the reason(s) for this increased mortality were not clear. In the present study, we investigated whether this difference in case fatality might be partially explained by RA patients receiving different rates of treatment with acute reperfusion or secondary prevention medications or both after the acute event.

We found that RA patients were substantially less likely to be treated with acute reperfusion after MI, and that this difference persisted after adjusting for type of MI, clinical setting of the MI, and prior MI (OR, 0.2; 95% CI, 0.05 to 0.6). We attempted to determine the explanation for this discrepancy by examining the charts for the reasons for failure to provide acute reperfusion in STEMI patients. Diagnostic delay was the reason for failure to treat in 11 (44%) of the 25 STEMI patients who did not receive acute reperfusion. Delayed presentation was more common in the RA patients, whereas a delay in confirming the diagnosis (after presentation) was more common in the controls. No identifiable reason was found for failure to treat in nine (36%) of the 25 STEMI patients who did not receive acute reperfusion.

Within the group of STEMI patients who did not receive acute reperfusion, the most obvious difference between the RA and control patients was in the frequency of contraindication to acute reperfusion. In one third of the RA STEMI patients who did not receive acute reperfusion, the reason recorded was the presence of a contraindication--in most cases, a preceding acute medical or surgical event. Similarly, in the overall cohort of 180 patients, a preceding medical illness or surgical event was more common in the RA patients than in the controls (31% versus 16%, respectively). Lichtman *et al*. [15] demonstrated in non-RA patients that the presence of a concurrent acute, severe, noncardiac condition at the time of MI was associated with lower rates of treatment with reperfusion therapy (even among patients who were "ideal candidates") and was associated with higher in-hospital mortality. Although the higher rate of concomitant illness in the RA patients in our study may have contributed to lower rates of acute reperfusion and secondary prevention therapy in this group, it is important to emphasize that the discrepancies persisted even with analysis adjusted for the clinical setting of MI.

Secondary prevention with medications such as aspirin, beta blockers, angiotensin-converting enzyme (ACE) inhibitors, and lipid-lowering agents is an important component of post-MI care [13]. A major finding of our study was that RA patients received lower rates of treatment with secondary prevention medications after MI. This was evident both in the total study cohort and in those patients who had experienced MI before the "MI admission." In the decade covered by our study, RA patients were 58% less likely to receive treatment with beta blockers and 79% less likely to receive treatment with lipid-lowering agents. Given that prompt implementation of reperfusion therapy and post-MI treatment with beta blockers and lipid-lowering agents are major determinants of outcome after acute MI, this lower rate of treatment after MI may help to explain why case-fatality rates are higher in RA than in the general population. It is unclear why RA patients were undertreated with secondary prevention medications; the discrepancy is not explained by differences in the prevalence of contraindication or serious comorbidity, because in these instances, the treatment was considered to have been provided. It is possible that concerns regarding toxicity, such as myositis, or exacerbating polypharmacy in the RA patients led to reluctance to prescribe additional medications.

Our study covered a 10-year period from 1995 to 2005, during which time the treatment of acute coronary syndromes evolved considerably. In the late 1990s and early 2000s, a significant shift occurred toward more-aggressive use of reperfusion therapy and routine prescription of lipid-lowering agents for secondary prevention of acute coronary syndromes (regardless of lipid levels) after publication of evidence supporting this practice [16-19]. Our study design, in which patients are matched for year of MI, compensates for changing practice over time. Having demonstrated lower rates of acute reperfusion and secondary prevention treatment in the RA patients, we were interested to explore the pattern of treatment over time. We found that for virtually all interventions, the RA patients received less-frequent treatment than did the controls, and that the differences persisted throughout all time periods. This suggests that the relative undertreatment of RA patients who experience MI is ongoing, despite the considerable literature relating to cardiovascular risk in RA.

This study has a number of potential limitations. We sampled only a small proportion of the RA patients in Victoria who experienced a hospital admission for MI over the 10-year study period. During the case-selection process, RA patients could have been missed if they were not coded as having RA during their hospital admission(s). The Victorian hospital coding standard requires that concurrent diagnoses that receive treatment during hospitalization be coded. Between 12 and 40 concurrent diagnoses are coded, depending on the year. It would therefore be expected that an RA patient whose medications were continued in hospital during the MI admission would be coded as RA, regardless of whether they had ever been admitted for RA. Misclassification would be more likely for patients with mild RA (not receiving any treatment) and may mean that only RA patients with more-severe disease were included in the study. Increased ascertainment of RA status in patients who experienced MI in the context of a rheumatoid-related admission is also possible. Such misclassification could mean that our sample is not representative of the full spectrum of RA patients and that our results may be relevant only to patients with more-severe disease. Our study included only metropolitan hospitals, so may not reflect practice in rural locations. Diagnosis of cardiovascular risk factors and other comorbidities was based on patient self-report and therefore may be inaccurate; however, the degree of inaccuracy is unlikely to be different between the RA and control groups. Furthermore, the presence or absence of the listed cardiovascular risk factors and comorbidities are not determinants of whether to provide acute reperfusion in the event of MI and thus do not act as confounders in the comparison. In one third of STEMI patients, no reason was ascertainable for not providing acute reperfusion treatment. It is possible that valid reasons existed for some or all of these episodes of "failure to treat," but that this rationale was not documented and therefore not apparent to the investigators. Again, it is unlikely that documentation omissions were distributed differently between RA and non-RA cases; therefore, bias is unlikely.

## Conclusions

In this retrospective study of 90 RA patients and 90 matched controls with acute MI, we found that the RA group received acute reperfusion and secondary prevention medications less frequently than did the control group. These discrepancies persisted despite adjustment for relevant clinical factors and were present within all time periods within the study duration. RA patients should be considered a high-risk group after MI and considered for acute reperfusion therapy along the same lines as the general population, in line with current best practice. In the absence of contraindications, RA patients should also receive recommended secondary prevention therapy after MI, in keeping with evidence-based guidelines for the general population.

## Abbreviations

ACE: angiotensin converting enzyme; ACR: American College of Rheumatology; CABGS: coronary artery bypass graft surgery; CI: confidence interval; DMARD: disease modifying anti-rheumatic drug; ICD: International Classification of Diseases; IDDM: insulin dependent diabetes mellitus; MI: myocardial infarction; NIDDM: non-insulin-dependent diabetes mellitus; NSAID: nonsteroidal anti-inflammatory drug; OR: odds ratio; PCA: percutaneous coronary angiography; PCI: percutaneous intervention; RA: rheumatoid arthritis; STEMI: ST-elevation myocardial infarction.

## Competing interests

The authors declare that they have no competing interests.

## Authors' contributions

SV conceived the study, coordinated the design and completion, supervised the chart-review process, performed the statistical analysis, and drafted the manuscript. CB and VS assisted in the design of the study and statistical analysis and helped to draft the manuscript. AA and IW assisted in the design of the study and helped to draft the manuscript. All authors read and approved the final manuscript.
